# Supplementary material for: Resolving the full spectrum of human genome variation using Linked-Reads
Source: Genome Res. 2019 Apr;29(4):635–45. doi: 10.1101/gr.234443.118 (PMC6442396; doi:10.1101/gr.234443.118)
Supplement: Supplemental Material [file supp_gr.234443.118_Supplemental_longranger-2.2.zip › longranger-2.2.tar.bz2!/lr2.2-public/lib/stan/cmdstan/stan/doc/api/html/argument__parser_8hpp_source.html]

Stan: src/stan/gm/arguments/argument\_parser.hpp Source File


|  |  |
| --- | --- |
|  | Stan  2.5.0  probability, sampling & optimization |


- Main Page
- Related Pages
- Namespaces
- Classes
- Files

- File List
- File Members

All Classes Namespaces Files Functions Variables Typedefs Enumerations Enumerator Friends Macros Pages

- src
- stan
- gm
- arguments

argument\_parser.hpp

Go to the documentation of this file.

1 #ifndef STAN\_\_GM\_\_ARGUMENTS\_\_ARGUMENT\_\_PARSER\_\_HPP

2 #define STAN\_\_GM\_\_ARGUMENTS\_\_ARGUMENT\_\_PARSER\_\_HPP

3

4 #include <string>

5 #include <vector>

6 #include <fstream>

7 #include <cstring>

8

9 #include <stan/gm/arguments/argument.hpp>

10 #include <stan/gm/arguments/arg\_method.hpp>

11 #include <stan/gm/error\_codes.hpp>

12

13 namespace stan {

14

15  namespace gm {

16

17  class argument\_parser {

18

19  public:

20

21  argument\_parser(std::vector<argument\*>& valid\_args)

22  : \_arguments(valid\_args),

23  \_help\_flag(false),

24  \_method\_flag(false) {

25  \_arguments.insert(\_arguments.begin(), new arg\_method());

26  }

27

28  int parse\_args(int argc,

29  const char\* argv[],

30  std::ostream\* out = 0,

31  std::ostream\* err = 0) {

32

33  if (argc == 1) {

34  print\_usage(out, argv[0]);

35  return error\_codes::USAGE;

36  }

37

38  std::vector<std::string> args;

39

40  // Fill in reverse order as parse\_args pops from the back

41  for (int i = argc - 1; i > 0; --i)

42  args.push\_back(std::string(argv[i]));

43

44  bool good\_arg = true;

45  bool valid\_arg = true;

46  \_help\_flag = false;

47

48  std::vector<argument\*> unset\_args = \_arguments;

49

50  while(good\_arg) {

51

52  if (args.size() == 0)

53  break;

54

55  good\_arg = false;

56  std::string cat\_name = args.back();

57

58  // Check for method arguments entered without the method= prefix

59  if (!\_method\_flag) {

60

61  list\_argument\* method = dynamic\_cast<list\_argument\*>(\_arguments.front());

62

63  if (method->valid\_value(cat\_name)) {

64  cat\_name = "method=" + cat\_name;

65  args.back() = cat\_name;

66  }

67

68  }

69

70  std::string val\_name;

71  std::string val;

72  argument::split\_arg(cat\_name, val\_name, val);

73

74  if (val\_name == "method")

75  \_method\_flag = true;

76

77  std::vector<argument\*>::iterator arg\_it;

78

79  for (arg\_it = unset\_args.begin(); arg\_it != unset\_args.end(); ++arg\_it) {

80  if ( (\*arg\_it)->name() == cat\_name) {

81  args.pop\_back();

82  valid\_arg &= (\*arg\_it)->parse\_args(args, out, err, \_help\_flag);

83  good\_arg = true;

84  break;

85  }

86  else if ( (\*arg\_it)->name() == val\_name) {

87  valid\_arg &= (\*arg\_it)->parse\_args(args, out, err, \_help\_flag);

88  good\_arg = true;

89  break;

90  }

91

92  }

93

94  if (good\_arg) unset\_args.erase(arg\_it);

95

96  if (cat\_name == "help") {

97  \_help\_flag |= true;

98  args.clear();

99  } else if (cat\_name == "help-all") {

100  print\_help(out, true);

101  \_help\_flag |= true;

102  args.clear();

103  }

104

105  if (\_help\_flag) {

106  print\_usage(out, argv[0]);

107  return error\_codes::OK;

108  }

109

110  if (!good\_arg && err) {

111

112  \*err << cat\_name << " is either mistyped or misplaced." << std::endl;

113

114  std::vector<std::string> valid\_paths;

115

116  for (size\_t i = 0; i < \_arguments.size(); ++i) {

117  \_arguments.at(i)->find\_arg(val\_name, "", valid\_paths);

118  }

119

120  if (valid\_paths.size()) {

121  \*err << "Perhaps you meant one of the following valid configurations?" << std::endl;

122  for (size\_t i = 0; i < valid\_paths.size(); ++i)

123  \*err << " " << valid\_paths.at(i) << std::endl;

124  }

125  }

126  }

127

128  if (\_help\_flag)

129  return error\_codes::OK;

130

131  if (!\_method\_flag)

132  \*err << "A method must be specified!" << std::endl;

133

134  return (valid\_arg && good\_arg && \_method\_flag) ?

135  error\_codes::OK : error\_codes::USAGE;

136  }

137

138  void print(std::ostream\* s, const std::string prefix = "") {

139  if (!s)

140  return;

141

142  for (size\_t i = 0; i < \_arguments.size(); ++i) {

143  \_arguments.at(i)->print(s, 0, prefix);

144  }

145

146  }

147

148  void print\_help(std::ostream\* s, bool recurse) {

149  if (!s)

150  return;

151

152  for (size\_t i = 0; i < \_arguments.size(); ++i) {

153  \_arguments.at(i)->print\_help(s, 1, recurse);

154  }

155  }

156

157  void print\_usage(std::ostream\* s, const char\* executable) {

158  if (!s)

159  return;

160

161  std::string indent(2, ' ');

162  int width = 12;

163  \*s << std::left;

164

165  \*s << "Usage: " << executable << " <arg1> <subarg1\_1> ... <subarg1\_m>"

166  << " ... <arg\_n> <subarg\_n\_1> ... <subarg\_n\_m>"

167  << std::endl << std::endl;

168

169  \*s << "Begin by selecting amongst the following inference methods"

170  << " and diagnostics," << std::endl;

171

172  std::vector<argument\*>::iterator arg\_it = \_arguments.begin();

173  list\_argument\* method = dynamic\_cast<list\_argument\*>(\*arg\_it);

174

175  for (std::vector<argument\*>::iterator value\_it = method->values().begin();

176  value\_it != method->values().end(); ++value\_it) {

177  \*s << std::setw(width)

178  << indent + (\*value\_it)->name()

179  << indent + (\*value\_it)->description() << std::endl;

180  }

181  \*s << std::endl;

182

183  \*s << "Or see help information with" << std::endl;

184  \*s << std::setw(width)

185  << indent + "help"

186  << indent + "Prints help" << std::endl;

187  \*s << std::setw(width)

188  << indent + "help-all"

189  << indent + "Prints entire argument tree" << std::endl;

190  \*s << std::endl;

191

192  \*s << "Additional configuration available by specifying" << std::endl;

193

194  ++arg\_it;

195  for (; arg\_it != \_arguments.end(); ++arg\_it) {

196  \*s << std::setw(width)

197  << indent + (\*arg\_it)->name()

198  << indent + (\*arg\_it)->description() << std::endl;

199  }

200

201  \*s << std::endl;

202  \*s << "See " << executable << " <arg1> [ help | help-all ] "

203  << "for details on individual arguments." << std::endl << std::endl;

204

205  }

206

207  argument\* arg(std::string name) {

208  for (std::vector<argument\*>::iterator it = \_arguments.begin();

209  it != \_arguments.end(); ++it)

210  if ( name == (\*it)->name() )

211  return (\*it);

212  return 0;

213  }

214

215  bool help\_printed() {

216  return \_help\_flag;

217  }

218

219  protected:

220

221  std::vector<argument\*>& \_arguments;

222

223  // We can also check for, and warn the user of, deprecated arguments

224  //std::vector<argument\*> deprecated\_arguments;

225  // check\_arg\_conflict // Ensure non-zero intersection of valid and deprecated arguments

226

227  bool \_help\_flag;

228  bool \_method\_flag;

229

230  };

231

232  } // gm

233

234 } // stan

235

236 #endif

237

stan::gm::argument\_parser::parse\_args

int parse\_args(int argc, const char \*argv[], std::ostream \*out=0, std::ostream \*err=0)

**Definition:** argument\_parser.hpp:28

stan::gm::argument\_parser::help\_printed

bool help\_printed()

**Definition:** argument\_parser.hpp:215

stan::gm::arg\_method

**Definition:** arg\_method.hpp:14

stan::gm::argument\_parser

**Definition:** argument\_parser.hpp:17

stan::gm::error\_codes::OK

**Definition:** error\_codes.hpp:11

argument.hpp

error\_codes.hpp

stan::gm::argument\_parser::arg

argument \* arg(std::string name)

**Definition:** argument\_parser.hpp:207

stan::gm::error\_codes::USAGE

**Definition:** error\_codes.hpp:12

stan::gm::list\_argument

**Definition:** list\_argument.hpp:12

stan::gm::argument\_parser::print\_usage

void print\_usage(std::ostream \*s, const char \*executable)

**Definition:** argument\_parser.hpp:157

stan::gm::argument\_parser::\_method\_flag

bool \_method\_flag

**Definition:** argument\_parser.hpp:228

stan::gm::argument::split\_arg

static void split\_arg(const std::string &arg, std::string &name, std::string &value)

**Definition:** argument.hpp:54

stan::gm::argument\_parser::argument\_parser

argument\_parser(std::vector< argument \* > &valid\_args)

**Definition:** argument\_parser.hpp:21

arg\_method.hpp

stan::gm::argument\_parser::\_arguments

std::vector< argument \* > & \_arguments

**Definition:** argument\_parser.hpp:221

stan::gm::argument\_parser::print\_help

void print\_help(std::ostream \*s, bool recurse)

**Definition:** argument\_parser.hpp:148

stan::gm::list\_argument::valid\_value

bool valid\_value(std::string name)

**Definition:** list\_argument.hpp:143

stan::gm::argument::name

std::string name() const

**Definition:** argument.hpp:26

stan::gm::argument\_parser::\_help\_flag

bool \_help\_flag

**Definition:** argument\_parser.hpp:227

stan::gm::list\_argument::values

std::vector< argument \* > & values()

**Definition:** list\_argument.hpp:158

stan::gm::argument

**Definition:** argument.hpp:12

stan::gm::argument\_parser::print

void print(std::ostream \*s, const std::string prefix="")

**Definition:** argument\_parser.hpp:138


---

[ Stan Home Page ]

*© 2011–2014,
Stan Development Team.*
